# Clinical and bi-genomic DNA findings of patients suspected to have mitochondrial diseases

**DOI:** 10.3389/fgene.2023.1191159

**Published:** 2023-06-12

**Authors:** Asuman Gedikbasi, Guven Toksoy, Meryem Karaca, Cagri Gulec, Mehmet Cihan Balci, Dilek Gunes, Seda Gunes, Ayca Dilruba Aslanger, Gokcen Unverengil, Birsen Karaman, Seher Basaran, Mubeccel Demirkol, Gulden Fatma Gokcay, Zehra Oya Uyguner

**Affiliations:** ^1^ Department of Pediatric Basic Sciences, Institute of Child Health Istanbul University, Istanbul, Türkiye; ^2^ Division of Pediatric Nutrition and Metabolism, Department of Pediatrics, Istanbul Faculty of Medicine, Istanbul University, Istanbul, Türkiye; ^3^ Department of Medical Genetics, Istanbul Faculty of Medicine, Istanbul University, Istanbul, Türkiye; ^4^ Department of Pathology, Istanbul Faculty of Medicine, Istanbul University, Istanbul, Türkiye

**Keywords:** mitochondrial diseases, mtDNA, bi-genomic sequencing, exome sequencing, differential diagnosis

## Abstract

**Background:** Mitochondrial diseases are the most common group of inherited metabolic disorders, causing difficulties in definite diagnosis due to clinical and genetic heterogeneity. Clinical components are predominantly associated with pathogenic variants shown in nuclear or mitochondrial genomes that affect vital respiratory chain function. The development of high-throughput sequencing technologies has accelerated the elucidation of the genetic etiology of many genetic diseases that previously remained undiagnosed.

**Methods:** Thirty affected patients from 24 unrelated families with clinical, radiological, biochemical, and histopathological evaluations considered for mitochondrial diseases were investigated. DNA isolated from the peripheral blood samples of probands was sequenced for nuclear exome and mitochondrial DNA (mtDNA) analyses. MtDNA sequencing was also performed from the muscle biopsy material in one patient. For segregation, Sanger sequencing is performed for pathogenic alterations in five other affected family members and healthy parents.

**Results:** Exome sequencing revealed 14 different pathogenic variants in nine genes encoding mitochondrial function peptides (*AARS2, EARS2, ECHS1, FBXL4, MICOS13, NDUFAF6, OXCT1, POLG*, and *TK2*) in 12 patients from nine families and four variants in genes encoding important for muscle structure (*CAPN3, DYSF,* and *TCAP*) in six patients from four families. Three probands carried pathogenic mtDNA variations in two genes (*MT-ATP6* and *MT-TL1*). Nine variants in five genes are reported for the first time with disease association: (*AARS2*: c.277C>T/p.(R93*), c.845C>G/p.(S282C); *EARS2*: c.319C>T/p.(R107C), c.1283delC/p.(P428Lfs*); *ECHS1*: c.161G>A/p.(R54His); c.202G>A/p.(E68Lys); *NDUFAF6*: c.479delA/p.(N162Ifs*27); and *OXCT1*: c.1370C>T/p.(T457I), c.1173-139G>T/p.(?).

**Conclusion:** Bi-genomic DNA sequencing clarified genetic etiology in 67% (16/24) of the families. Diagnostic utility by mtDNA sequencing in 13% (3/24) and exome sequencing in 54% (13/24) of the families prioritized searching for nuclear genome pathologies for the first-tier test. Weakness and muscle wasting observed in 17% (4/24) of the families underlined that limb-girdle muscular dystrophy, similar to mitochondrial myopathy, is an essential point for differential diagnosis. The correct diagnosis is crucial for comprehensive genetic counseling of families. Also, it contributes to making treatment-helpful referrals, such as ensuring early access to medication for patients with mutations in the TK2 gene.

## Introduction

Mitochondrial diseases (MDs), caused by mutations in either nuclear or mitochondrial genomes (mtDNA), are the most common group of inherited metabolic diseases and affect about 1 in 5,000 in the population (Stenton and Prokisch, 2020; Orsucci et al., 2021). High clinical and genetic heterogeneity, overlapping with neuromuscular diseases, is observed due to pathological changes in genes encoding complex energy metabolism pathways in organs with high energy requirements, such as muscles, the brain, and the heart. Clinical characteristics, biochemical and enzymatic analyses, brain imaging, and pathomorphological anomalies in muscles and fibroblasts are all assessed for clinical diagnosis (Rahman, 2020; Fernandez-Eulate et al., 2022).

Unique characteristics of the mitochondrial genome, such as heteroplasmy, maternal inheritance, and dynamic organelle structure involving more than 1,500 functional peptides encoded by nuclear DNA, complicate the molecular diagnosis of MDs (Schlieben and Prokisch, 2020). The underlying genetic origin may be secondary to pathogenic variants in one of the nuclear genes involved in the mitochondria’s transcription, translation, or function, concurrently small and gross pathogenic alterations in the mtDNA. The majority of the mitochondrial proteome is encoded by the nuclear genes. To date, 320 out of 1,136 MD-associated genes are proven to be disease-associated genes (Rath et al., 2021). A total of 96 disease-related variants are reported in 37 genes in the mitochondrial genome in pathogenic mutations confirmed using Mitomap (Lott et al., 2013).

Next-generation sequencing (NGS) technology allows whole-genome sequencing (WGS) or whole-exome sequencing (WES), including mtDNA. Based on clinical and genetic heterogeneity and unknown incidences of mitochondria-related diseases in different cohorts, non-targeted exome sequencing provides powerful access to molecular genetic diagnosis. NGS technology led to a paradigm shift in the differential diagnosis of MDs, altering the invasive function-to-gene approach to a non-invasive approach via the “genetics-first.” In parallel, since MDs are under the control of a dual genome, the classification of MDs has evolved from clinical and biochemical designations to a gene-based direction organized under the disease of mtDNA mutations and Mendelian mitochondrial disorders (Lightowlers et al., 2015). Despite numerous obstacles, motivation for providing molecular diagnosis in MDs is high because the clinical management of the disease, future care planning, and providing pre-implantation genetic diagnostic opportunities and relapse risk predictions are valuable for patients and their families. Moreover, definitive molecular diagnosis allows for replacing a critical cofactor for disease recovery or provides target-specific treatment such as restriction of a diet component. This study presents the results of dual-genome analysis toward diagnosing patients with a preliminary assessment of MDs with clinical findings, imaging, and biochemical values.

## Materials and methods

### Participants and clinical evaluations

The study group comprised 24 probands, six affected family members (families 1, 2, 6, 13, 16, and 18), and 29 healthy individuals [one healthy sibling from each of four families (families 5, 7, 8, and 9) and 25 healthy parents] from 24 unrelated families formed the study group. The participants provided consent to participate.

All affected individuals were suspected to have mitochondrial diseases (MDs) in the differential diagnosis according to clinical, radiological, and laboratory findings. Clinical genetic examination of patients is recorded, including radiological and laboratory findings with detailed medical history. Patients were evaluated at the genetic outpatient clinic, and a three-generation pedigree was drawn.

### Radiological evaluations

Central nervous system findings, nonvascular distribution of stroke-like lesions, diffuse white matter damage, bilateral gray matter, basal ganglia, and brain stem involvement have been evaluated from the magnetic resonance imaging (MRI) findings of patients. Electromyography (EMG), electroencephalogram (EEG), and echocardiography (ECG) reports were examined.

### Biochemical tests

The Carnitine–acylcarnitine profile was assessed by tandem mass spectrometry (MS/MS) and quantitative amino acid analyses, and organic acid analysis results were assessed by liquid and gas chromatography–mass spectrometry (LC and GC-MS). Blood lactate levels, alanine levels elevated secondary to the transamination of pyruvate, and increased excretion of urinary organic acids as tricarboxylic acid (TCA) cycle intermediates such as lactate and malate, succinate, 2-oxoglutarate, and fumarate were recorded. Creatine kinase (CK), aspartate aminotransferase (AST), and alanine aminotransferase (ALT) levels indicating muscle and liver involvement were documented. Ketone bodies like 3-OH-butyrate and acetoacetate were evaluated.

### Histopathological tests

A microscopic examination of the tissue was performed. Muscle biopsy samples inside gauze pads were frozen in liquid nitrogen, and sections were obtained using a cryotome device at −80°C. The 8-μm sections were subjected to hematoxylin and eosin (HE), modified Gomori trichrome (MGT), PAS, oil Red O (ORO), myophosphorylase, and oxidative enzyme (NADH-TR, SDH, COX, and COX/SDH) stains for examination.

### Genetic tests

Genomic DNA, together with mtDNA, was isolated from peripheral blood lymphocytes. A stepwise approach was used for genetic investigation. The initial test was mtDNA sequencing by an in-house developed fragmentation-based methodology. Patients with unidentified heteroplasmic or homoplasmic causative variant associations with mtDNA were underwent a second analysis step for exome sequencing with either single, paired, or trio investigation.

### MtDNA sequencing

Four primer pairs, coupling on the flanking regions, were designed and used with the Thermo Scientific™ Phusion High-fidelity DNA Polymerase enzyme to amplify the mtDNA (16.569 bp) with four overlapping sizes, 5,077, 4,393, 5,465, and 5,540 bp. Amplified mtDNA samples are purified using the Roche High Pure PCR Product Purification Kit. The fragmentation process was performed using the Ion Xpress™ Plus Fragment Library Kit (Kat. No. 4471269). The library length was selected as 400 bp. Fragmentation was performed to maintain a median fragment size of 350–450 bp. The Ion 510 ™ Kit (Kat. No. A34461) was used to load samples to chips, the template was prepared in the Ion Chef ™ System, and sequencing was performed in the Ion S5 ™ System.

### Exome sequencing

The Ion AmpliSeq™ Exome RDY Kit 1 × 8™ (Cat. No. A38262) was used to prepare exome libraries for sequencing genomic DNA. Barcoded libraries were formed using the Ion Xpress™ Barcode Adapter Kits™ (Cat. No. 4474517; whole adapter set between 1 and 96). Barcoded libraries were loaded in a single ion chip, and data were transferred to Torrent server software following sequencing.

### Variant analysis

Data were automatically transferred to Ion Reporter™ software using Torrent Suite™ with the IonReporterUploader. The Browser Extensible Data (BED) file was added to the target and hotspot regions, and quality parameters were maintained in the recommended range. Each patient was analyzed with the analysis flow chart formed in the Ion Reporter™ System. Variants are viewed using the Integrative Genomics Viewer (IGV). The revised Cambridge Reference Sequence (rCRS) (GenBank access no: NC_012920.1), containing 16,569 base pairs, is used to determine mtDNA variants, and the Mitomap database was used to investigate the definitive pathogenic changes. Exome sequence variants with a minor allele frequency of <0.1% are classified according to the ACMG (Richards et al., 2015). In the data analysis, genes reported to be related to mitochondrial diseases (MitoCarta 3.0) are prioritized (Rath et al., 2021). Disease association of the exome variants is searched from the literature reports OMIM (Hamosh et al., 2005), ClinVar (Landrum et al., 2018), and GeneCards (Stelzer et al., 2016). Deleteriousness of the substitutions is scored by Combined Annotation-Dependent Depletion (CADD, 1.6) using the online server UCSC Browser for CADD scores in the phenotypes and literature track of the human gene (Rentzsch et al., 2019) and for deletion from the web server provided in the Mutation Significance Cutoff (MSC) of human genes (Itan et al., 2016). Domain/regions are defined according to UniProt (Dogan et al., 2016). The Human Splicing Finder is used to investigate the impact of alteration on exonic splicing silencer (ESS) and exonic splicing enhancer (ESE) sites (Desmet et al., 2009). Variants suspected to be pathogenic and clinically related are confirmed with by Sanger sequencing on ABI3500, followed by segregation in the families.

### RNA investigation

The total RNA was extracted from a peripheral blood sample’s lymphocytes or a muscle biopsy using the TRIzol manufacturer protocol (Invitrogen). mRNA was converted to cDNA using 1 μg of the total RNA per reaction, using a reverse transcriptase protocol with a random hexamer (Bio-Rad iScript). Sequencing primer pairs are designed to couple flanking coding exons of the target.

## Results

### Study group

The demographic characteristics of 30 affected patients from 24 families, clinical, laboratory, radiology, and muscle histochemistry findings are given in Table 1. Of the cohort, four (13%; three females and one male) were adults (31, 7 ± 8, 9), and 26 (87%; 13 females and 13 males) were under 18 years of age (9, 2 ± 5, 7 years).

**TABLE 1:** Clinical and laboratory findings of patients (A and B). Patient numbers with a sibling relationship are shown in bold letters. A. Patients identified with pathogenic variants that are associated clinically. B. Patients without any associated variant.

Demographic Data				Clinical Findings					Laboratory results				Muscle Biopsy evaluations			Radiologic images	Diagnostic ID
FPG	AoD	AoO	Cp	MF	NF	CF	GF	OF	Lac	CK	QAa	UOA	RR	M/MM	EA	Cranial MRI	OMIM
A																	
F-1	27	15	+	MyoP	-	-	+	+	↑	↑	NS	NS	+	MM	NA	N	# 609560 MITOCHONDRIAL DNA DEPLETION MYOPATHY
**P-1**																	
F																	
F-1	17	13	+	MyoP	-	-	+	+	↑	↑	NS	NS	+	MM	NA	N	# 609560 MITOCHONDRIAL DNA DEPLETION MYOPATHY
**P-2**																	
F																	
F-2	17	5	+	MyoP	-	-	-	+	↑	↑	NS	NS	+	MM	NA	N	# 258450 PEO, WITH MITOCHONDRIAL DNA DELETIONS
**P-3**																	
M																	
F-2	15	6	+	MyoP	-	-	-	+	↑	↑	NS	NS	+	MM	NA	N	# 258450 PEO, WITH MITOCHONDRIAL DNA DELETIONS
**P-4**																	
F																	
F-3	3	New born	-	MyoP	+	-	-	-	↑	↑	NS	↑	+	MM	NA	T2-weighted hyperintensities in dentate nucleus, thalamus, and periventricular deep white matter.	# 614924 COMBINED OXIDATIVE PHOSPHORYLATION DEFICIENCY 12
P-5																	
F																	
F-4	9	0,3	-	MyoP	+	-	-	+	↑	-	NS	NS	-	M	NA	T2-weighted hyperintensities in deep cerebral white matter	# 614096 COMBINED OXIDATIVE PHOSPHORYLATION DEFICIENCY 8
P-6																	
M																	
F-5	16	4	-	DysT	+	-	-	-	↑	N	NS	NS	-	NS	CxIV ↓	Decreased tissue volume in the putamen and caudate nucleus volume and pathological signal changes	# 618239 MITOCHONDRIAL COMPLEX I DEFICIENCY, NUCLEAR TYPE 17; MC1DN17
P-7																	
M																	
F-6	12, 10	0,3	-	DysT	+	-	-	-	N	N	NS	NS	NA	NA	NA	T2-weighted hyperintensities in the putamen and caudate nucleus	# 616277 MITOCHONDRIAL SHORT-CHAIN ENOYL-CoA HYDRATASE 1 DEFICIENCY
**P-8**, **P-9**																	
M, M																	
F-7	2	1	-	HypoT	+	-	+	-	↑	N	NS	↑	-	NS	CxIV↓	Cortical ischemic lesions and cerebellar atrophy	# 245050 SUCCINYL-CoA:3-OXOACID-CoA TRANSFERASE DEFICIENCY
P-10																	
M																	
F-8	8	1	+	HypoT	+	+	+	-	↑	N	NS	NS	-	MM	CxIV↓	Encephalopathic lesions involving white matter, deep gray nuclei, and brainstem	# 615471 MITOCHONDRIAL DNA DEPLETION SYNDROME 13
P-11																	
F																	
F-9	1	New born	+	MyoP	+	+	+	+	↑	↑	↑	↑	-	NS	NA	In T2W sagittal seq, atrophy of the cerebellum, brainstem, and corpus callosum, T2W hyperintensities periventricular deep white matter	# 618329 COMBINED OXIDATIVE PHOSPHORYLATION DEFICIENCY 37
P-12																	
M																	
F-10	11	6	-	MyoP	CA	-	-	-	↑	N	NS	NS	NS	MM	-	T2W hyperintense in the bilateral caudate nucleus, subthalamic nucleus, cerebellar hemisphere	* 516060 ATP SYNTHASE 6; MTATP6, COMPLEX V, ATP SYNTHASE
P-13																	
F																	
F-11	13	4	-	MyoP	CA	-	-	-	↑	N	NS	NS	NS	M	-	T2-weighted hyperintensities in basal ganglia	* 516060 ATP SYNTHASE 6; MTATP6, COMPLEX V, ATP SYNTHASE
P-14																	
F																	
F-12	9	8	-	MyoP	+	+	-	-	↑	↑	NS	NS	+	MM	-	T2-weighted hyperintensities in periventricular deep white matter.	* 590050 TRANSFER RNA, MITOCHONDRIAL, LEUCINE 1
P-15																	
F																	
F-13	19	12	+	MyoP	-	-	-	-	N	↑↑	NS	NS	-	MM	NA	N	# 253600 MUSCULAR DYSTROPHY, LIMB-GIRDLE, AUTOSOMAL RECESSIVE 1
**P-16**																	
M																	
F-13	16	13	+	MyoP	-	-	+	-	N	↑↑	NS	NS	-	MM	NA	N	# 253600 MUSCULAR DYSTROPHY, LIMB-GIRDLE, AUTOSOMAL RECESSIVE 1
**P-17**																	
M																	
F14	19	6	+	MyoP	-	-	-	-	-	↑↑	NS	NS	-	NS	NA	N	# 253600 MUSCULAR DYSTROPHY, LIMB-GIRDLE, AUTOSOMAL RECESSIVE 1
P-18																	
M																	
F-15	42	22	+	MyoP	-	+	-	-	-	↑↑	NS	NS	-	MM	NA	N	# 253601 MUSCULAR DYSTROPHY, LIMB-GIRDLE, AUTOSOMAL RECESSIVE 2
P-19																	
F																	
F-16	35, 21	6	+	MyoP	-	-	-	+	-	↑↑	NS	NS	-	M	NA	N	# 601954 MUSCULAR DYSTROPHY, LIMB-GIRDLE, AUTOSOMAL RECESSIVE 7
**P-20**																	
**P-21**																	
M, M																	
B																	
F-17	15	12	-	MyoP	-	-	-	+	-	-	NS	NS	+	MM	NA	N	None
P-22																	
F																	
F-18	6	1	+	MyoP	-	-	-	+	↑	↑↑	NS	↑	-	NS	mCx↓	N	None
**P-23**																	
F																	
F-18	3	0,6	+	MyoP	-	-	-	+	-	↑↑	NS	↑	NA	NA	NA	N	None
**P-24**																	
M																	
F-19	1	New born	+	HypoT	+	-	+	-	↑	↑	NS	↑↑	-	M	NA	T2-weighted hyperintensities in deep white matter.	None
P-25																	
M																	
F-20	4	1	+	HypoT	+	-	-	-	-	-	NS	NS	-	M	CxIV↓	Cranial MRI and MRS results were suggestive for MD	None
P-26																	
F																	
F-21	1	New born	+	HypeT	+	-	-	+	↑	↑	NS	↑	NA	NA	NA	Thinning of the corpus callosum	None
P-27																	
F																	
F-22	4	0,6	-	HypoT	+	-	-	-	-	-	NS	NS	-	M	-	Delayed myelination, thinning of the corpus callosum	None
P-28																	
F																	
F-23	11	1	-	HypoT	+	-	-	+	-	-	NS	NS	-	M	CxIIV ↓	Cerebral cerebellar atrophy	None
P-29																	
F																	
F-24	4	3	+	HypoT	+	-	-	+	↑	-	NS	NS	NA	NA	NA	T2-weighted hyperintensities in the putamen and caudate nucleus	None
P-30																	
M																	

F: family number; P: patient number; F: female; M: male; MD: mitochondrial disease; NA: not applicable; NS: non-specific; N: normal; MRI: magnetic resonance imaging; MRS: magnetic resonance spectrum.

The age of the onset of disease in the cohort varied from birth to 22 years. Consanguineous marriage was observed in 13 families (54%). Four patients had an affected sibling, and two had an affected cousin.

### Clinical findings

Muscular system findings were revealed in all patients (100%), and central nervous system findings were present in 17 (17/30), patients. In addition, eleven patients had ptosis or progressive external ophthalmoplegia (11/30), seven patients had abnormal liver function or gastrointestinal system findings (7/30), and four patients had cardiomyopathy (4/30).

### Laboratory findings

Of the 30 patients, 16 had elevated lactic acid levels (16/30, 53%), and 16 had elevated creatine kinase (CK) levels (16/30, 53%). Quantitative amino acid analysis revealed no specific findings except elevated alanine in one patient. Urine organic acid analysis revealed mitochondrial disease-related excretion in seven patients (7/30, 23%). Histopathological evaluation of the 24 muscular biopsy samples revealed mitochondrial myopathy (MM) findings in 13 patients (13/24, 54%), seven of whom had ragged red fibers (7/24, 29%). Others were reported to have mild myopathic changes (11/24, 46%). Complex enzyme activity was reported in only six patients from six families, isolated complex IV enzyme deficiencies in five (P7, 10, 11, 26, and 29), and multiple complex enzyme deficiencies in one (P23). Cranial MRI studies revealed pathological findings at specific sites consistent with mitochondrial disease in 17 patients (P5–15 and P25–30) (17/30, 57%), and no evidence of pathology was found in 13 patients (P1–4 and P16–24) (13/30, 43%) (mitochondrial depletion syndrome *n* = 4, LGMD *n* = 6, and three undiagnosed cases).

### Molecular analysis

**FIGURE 1:**
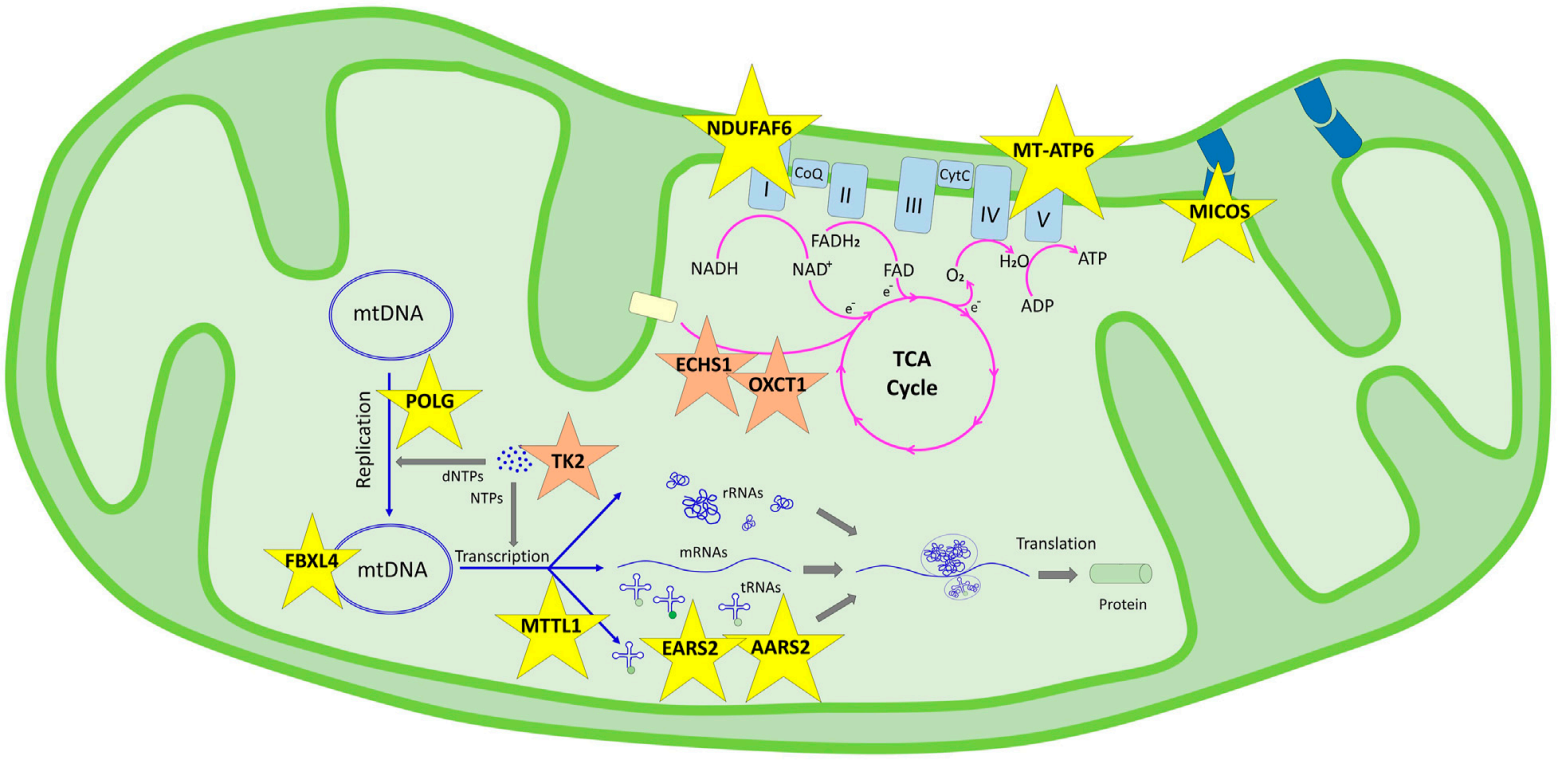
Schematic representation of the genes determined in this study and their pathways in the mitochondrial functions according to KEGG. Genes in the genetic information processing pathway are shown in yellow stars in two subgroups: translation/aminoacyl-tRNA biosynthesis (*AARS2*, *EARS2*, and *MT-TL1*) and mitochondrial biogenesis (*FBXL4*, *NDUFAF6*, *MICOS13*, *POLG*, and *MT-ATP6*). Genes in the metabolism pathway are shown in orange stars in three subgroups: nucleotide metabolism (*TK2*), carbohydrate metabolism (*OXCT1*), and fatty acid elongation (*ECHS1*).

A total of 14 variants in nine nuclear genes (*AARS2, EARS2, ECHS1, FBXL4, MICOS13, NDUFAF6, OXCT1, POLG,* and *TK2*) in nine probands and two variants in two mtDNA genes (*MT-ATP6* and *MT-TL1*) in three probands were disclosed (Table 2). The association of those genes in the related pathways involved in mitochondrial function are shown in Figure 1. The inheritance model was confirmed by parental DNA testing for all variants identified in the nuclear genome. Three healthy siblings from families 5, 7, and 9 did not carry the related variant, while the healthy sibling of P11 of family 8 was found to be a heterozygous carrier. Nine variants were associated with MDs in this study submitted to ClinVar, shown in bold letters in Table 2. The time for molecular diagnosis ranged between 1 year and 29 years (median of 9 years).

**TABLE 2:** Detail descriptions of the variants identified in patients. ACMG classifications of variants are performed by the Franklin website, accessed 21 April 2023 (https://franklin.genoox.com) based on population data, functional data, *in-silico* predictions, and reputable source data as availability.

Family number	Patient numbers	Gene; Transcript; Peptid ID	Variant in nucleotide	Variant in peptide	Domain	Zygosity	dbSNP	CADD scores	ClinVar	ACMG classification/criterias fulfilled
1	**1,2**	*TK2;* NM_004614.5; NP_000061.1	c.323C>T	p.(T108M)	dNK	Hom.	rs137854431	25.5	VCV001442595.2	Pathogenic/PM2, PM1, PP2, PP3, PP5
2	**3,4**	*POLG;* NM_001126131.1;NP_001119603.1	c.911T>G	p.(L304L)	DNApol_Exo	Hom.	rs121918044	28.5	VCV000013497.40	Pathogenic/PM1, PP2, PM2, PP3,PP5
3	5	*EARS2;* NM_001083614.2;NP_001077083.1	**c.[319C>T];[1283delC]**	p.([p.R107C][P428Lfs*3])	tRNA-synt_1c; -	Comp. het.	rs1355685453; -	23;22.4	**SCV001423134.1** VCV000973188.2 **SCV001423135.1** VCV000973189.2	Pathogenic/PM2, PM5, PM1, BP4 Pathogenic/PVS1, PM2, PP5
4	6	*AARS2;* NM_020745.4;NP_065796.2	**c.[277C>T];[845C>G]**	**p.([R93Ter];[S282C])**	-:-	Comp. het.	rs760920084;-	37;28.3	**SCV001571651.1** VCV001064699.2 **SCV001571652.1** VCV001064700.2	Pathogenic/PVS1, PM2, PP5; Pathogenic/PM2, PP3, PP5
5	7	*NDUFAF6*; NM_152416.4; NP_689629.2	**c.[479delA];**[420+784C>T ]	p.([N162Ifs*27];[?])	?;-	Comp. het.	-;rs749738738	?; 16.04	**SCV001571649.1** VCV001064697.2 VCV000929495.4	Pathogenic/PVS1, PM2, PP5; Likely Pathogenic/PM2, PP5
6	**8,9**	*ECHS1*; NM_004092.4; NP_004083.3	**c.[161G>A];[202G>A]**	**p.([R54H];[E68K])**	-;-	Comp. het.	rs375266808 rs1276839756	26.8;24.9	**SCV003804637 SCV003804638**	Pathogenic/PM5, PM1, PP2, PP5; VUS/PM2, PM1, PP2, PP3
7	10	*OXCT1*; NM_000436.4; NP_000427.1	**c.[1370C>T];[1173-139G>T]**	**p.([T457I];[?])**	-;NA	Comp. het.	rs369643387 rs145673650	25.6;6.189	**SCV001571647.1** VCV001064695.2 **SCV001571646.1** VCV001064694.2	Likely Pathogenic/PM2, PP5; Likely Pathogenic/PM2/BP7, PP5
8	11	*FBXL4*; NM_001278716.2; NP_001265645.1	c.1444C>T	p.(R482*)		Hom.	rs398123061	**24.9**	VCV000066093.19	Pathogenic/PM2, PP2, PP5; ​
9	12	*MICOS13*; NM_205767.3; NP_991330.1	c.260-2A>G	p.(?)	NA	Hom.	rs1064797230	33	VCV000425157.15	Pathogenic/PVS1, PM2, PP3
10, 11	13, 14	*MT-ATP6*	m.8993T>C	p.(L156P)	transmembrane	Homoplasmy	rs199476133	-	VCV000009642.28	Pathogenic/PS1, PM5, PM2, PP3, PP5
12	15	*MT-TL1*	m.3243A>G	NA	NA	Heteroplasmy (50% blood 85% muscle)	rs199474657	-	VCV000009589.46	Likely Pathogenic/PM2, PP5
13	**16,17**	*CAPN3*; NM_000070.3; NP_000061.1	c.550delA	p.(T184Rfs*36)		Hom.	rs80338800		VCV000017621.58	Pathogenic/PVS1, PM2, PP5
14	18		c.1992+1G>T	p.(?)	NA	Hom.	rs863224961	36	VCV000217154.44	Pathogenic/PVS1, PM2, PP5
15	19	*DYSF*; NM_003494.4; NP_003485.1	c.5767+1G>A	p.(?)	NA	Hom.	rs756689063	34	VCV000597680.10	Pathogenic/PVS1, PM2, PP5
16	**20,21**	*TCAP*; NM_003673.4; NP_003664.1	c.90_91del	p.(S31Hfs*11)		Hom.	rs1555606976		VCV000446463.22	Pathogenic/PVS1, PM2, PP5

Patients (siblings) from the same family, novel alterations, and ClinVar submission numbers are shown in bold letters. Human gene names are written in capital letters and italics. VUS, variant of unknown significance; Hom., homozygous; Comp. het., Compound heterozygous; dNK, Deoxynucleoside kinase; DNApol_Exo, DNA mitochondrial polymerase exonuclease domain; tRNA-synt_1c, tRNA synthetases class I (E and Q), catalytic domain; PM, pathogenic moderate; PP, pathogenic supporting; PVS, pathogenic very strong; BP, benign supporting.

**FIGURE 2:**
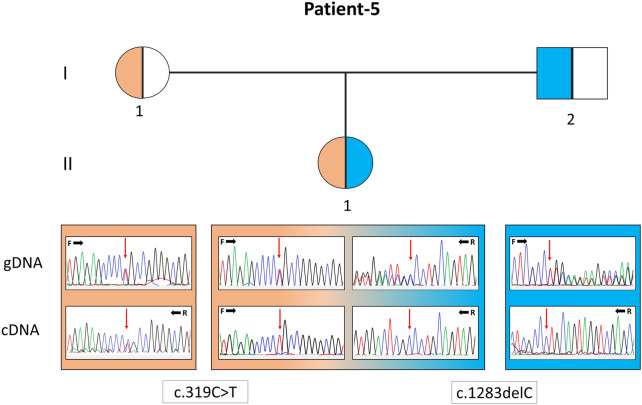
Pedigree of patient 5 with the *EARS2* gene variants. Electropherograms of the sequences from the genomic DNA (gDNA) and complementary DNA (cDNA) are presented. The presence of only the allele carrying the missense variant in the patient and only the normal allele in her father indicates that the deletion-type mutation was subjected to mRNA decay.

In P22, a homozygous c.260C>T/p.(T87I) rs144000161 variant in the *COX10* gene was identified and classified as a variant of unknown significance (VUS), and its association with the disease was excluded by detecting this change in the homozygous form in the healthy sibling. Electropherograms of the two different variants detected in the *EARS2* gene on cDNA synthesized from the total RNA obtained from peripheral blood samples in P5 (II:1) and her parents (I:1 and I:2) showed that the inheritance of the allele with a frameshift to early stop underwent mRNA decay (Figure 2). The molecular genetic analysis showed MDs in 15 patients from 12 families. On identifying the relevant variants in the *CAPN3, DYSF*, and *TCAP* genes, the diagnosis was oriented toward limb-girdle muscular dystrophy (LGMD) in six patients from four families. The molecular basis in eight families (8/24, 33%) could not be determined (Table 1).

## Discussion

The involvement of diverse organs and various ages of onset and severity complicate the molecular pathologies underlining the MDs. Serious obstacles resulting from the impaired interactions among the molecules required for cellular homeostasis and metabolism can be grouped under complex pathways involved in mitochondrial function and integrity due to pathogenic changes in genes encoded by either mtDNA or genomic DNA, resulting in severe diseases with separately grouped organ involvements, age of onset, and heterogeneous phenotype. These interactions are classified under manifolds of pathways (Kyoto Encyclopedia of Genes and Genomes, KEGG) like metabolism, genetic information processing, environmental information processing, cellular processing, organizational systems, and drug interactions (Kanehisa et al., 2023).

In our cohort, mitochondrial pathway associations were defined for 11 genes; nine were genomic and two were mitochondrial DNA genes, enlightening accurate diagnosis in 12 of our families. Although extensive interactions overlap in pathways, we discuss the patient’s findings under those pathway involvements (Figure 1).

### Genetic information processing/translation/aminoacyl-tRNA biosynthesis (*AARS2, EARS2,* and *MT-TL1*)

Human diseases caused by mitochondrial translation defects may arise from pathogenic variations in mitochondrial tRNA, mitochondrial aminoacyl-tRNA synthetase, mitochondrial rRNA, and mitochondrial ribosomal protein-encoding genes (Webb et al., 2020). Most infantile-onset mitochondrial encephalopathies that are genetically unidentified may have prominent biochemical characteristics related to multiple respiratory chain complex defects, its subunits encoded either by the mtDNA or, for the majority, by the nuclear DNA. Aminoacyl-tRNA synthetases (ARSs) are a group of structures recently reported to be involved in the peripheral nervous system, either with loss- or gain-of-function mutations (Boczonadi et al., 2018). *AARS2* and *EARS2* encode mitochondrial aminoacyl-tRNA synthetases (mt-ARSs), essential for protein synthesis in the mitochondria and generation of oxidative phosphorylation (OXPHOS) system components. Combined OXPHOS deficiency (COXPD) is the most common of biallelic diseases associated with mitochondrial-ARSs (Turvey et al., 2022). *MT-TL1* is one of the 22 mt-tRNAs encoded by the mitochondrial genome and is primarily responsible for delivering amino acids to the nascent polypeptide chain during mitochondrial protein translation. In our cohort, P6, 5, and 15 were associated with pathogenic alteration in the *AARS2*, *EARS2,* and *MT-TL1* genes. P6 was a 9-year-old male with seizures, myopathy and ophthalmological findings whose findings started at the postnatal third month and carried a compound heterozygous variant in the *AARS2* gene. P5, with compound heterozygous pathogenic variants in the *EARS2* gene, was a 1.5-year-old baby girl with early-onset hypoglycemic seizures, inability to hold her head, hypotonia, epilepsy, imaging, and laboratory findings. Cranial MRI revealed symmetrical T2A pathological signal intensity increase in bilateral dentate nuclei, thalamus, and periventricular deep white matter. The patient and, as a carrier, her father showed mRNA decay of the deletion allele (c.1283delC). In a study with 12 patients with various *EARS2* gene mutations, all had similar MRI findings (Steenweg et al., 2012), suggesting that cranial MRI may be more accountable evidence than standard biochemical studies for the discrimination of ARS-associated diseases. Although several MRI findings highly suggest specific ARS mutations, no MRI pattern is common to all patients (Roux et al., 2021). P15 was a 9-year-old female with the most prevalent MELAS (Mitochondrial encephalomyopathy, lactic acidosis and stroke-like episodes) variant in the *MT-TL1* gene (m.3243A>G), found in 50% and 85% heteroplasmic cells in her blood and muscle cells, respectively. Among MDs caused by defects in mt-tRNAs, m.3243A>G is the most common and accounts for ∼80% of all mt-tRNA-related MDs, and the relationship between pathogenic mt-tRNA levels and the clinical phenotype is not correlated (Richter et al., 2021). Mammalian mt-tRNA genes have accumulated more mutations than their cytoplasmic counterparts during evolution. It has been proposed that the induced adaptation of cognate mitochondrial ARSs compensates for the structural complexity in mammalian mt-tRNAs. This interaction is critical for understanding the disease and severity of mt-tRNA mutations.

### Genetic information processing/mitochondrial biogenesis (*FBXL4, NDUFAF6, MICOS13, POLG,* and *MT-ATP6*)

The *FBXL4* gene encodes a subunit of modular E3 ubiquitin ligase as a regulator of mitochondrial biogenesis, and *NDUFAF6* encodes a subunit of the complex-1 factor of the mitochondrial respiratory chain complex assembly factors grouped under major mitochondrial quality control factors (KEGG). P11 has homozygous missense variation (c.1444C>T/p.(R482W)) in the FBXL4 gene and the variants that were previously described (Gai et al., 2013). Clinical findings attributed to this variant in the literature were highly heterogeneous in the three affected male siblings; the first child died due to a severe lactic acidosis attack at 4 years of age; the second was alive at 9 years of age and had mild facial dysmorphism with severe intellectual disability, intermittently elevated plasma lactate levels, and exercise intolerance; and the third was 6 months old with neonatal depression at delivery and attacks of severe lactic acidosis. However, growth parameters and facial appearance were normal. P11, at the 26th gestational age, presented developmental delay. In her follow-ups at 15 months of age, she had lactic acidosis and elevated blood alanine levels, and organic acid analysis revealed excretion consistent with mitochondrial disease. Muscle biopsy analysis revealed OXPHOS enzyme deficiency, and histopathology was reported as lipid storage myopathy. She was under follow-up of cardiology due to hypertrophic cardiomyopathy. She is currently 9 years old. Her Denver developmental screening test showed a 50% delay in the developmental milestones, her verbal ability was restricted, and she can only say a few sentences with speech therapy. She also had mild dysmorphic facial characteristics.

P7 with compound heterozygous variants in the *NDUFAF6* gene-encoding factor 6 of the NADH dehydrogenase complex I was predicted to have dysfunctional highly preserved mitochondrial proteins among eukaryotes that play an essential role in the early combining stage of the mitochondrial respiratory chain. *NDUFAF6* pathologies in the literature are related to a type of Leigh syndrome without cognitive involvement or seizures, manifesting with isolated cerebellar or extrapyramidal symptoms depending on the affected structures that onset with the loss of abilities in childhood between 1 and 5 years of age and staying stable or progresses slowly (Catania et al., 2018). P7’s clinical findings were similar to those reported in the literature. He showed a loss of abilities developed after 3 years of age, inability to walk and talk, dystonic contractures, scoliosis, and contracture in his hands.

P12 was a 1-year-old male identified with homozygous acceptor splice site alteration (c.260–2A>G) in intron 3 of the *MICOS13* gene. The *MICOS13* gene encodes a component of the MICOS complex, a large protein complex of the mitochondrial inner membrane that forms contact with the outer membrane. Before his accurate diagnosis, he had been treated in the neonatal intensive care unit for 1 month with a presumptive diagnosis of metabolic disease and symptoms of lack of sucking on the third postnatal day, respiratory distress, jaundice, severe metabolic acidosis, and hyperammonemia findings. He passed away at 15 months of age due to severe metabolic acidosis and respiratory failure. The *MICOS13* variant identified was previously associated with combined oxidative phosphorylation deficiency in a baby girl whose initial signs were observed later than ours at 6 months. mRNA investigation obtained from her fibroblast culture cells showed two aberrant splice sites, one with 21 bp reduced size of exon 4, and the second was a frameshift by the deletion of 44 bp of exon 4 with a premature stop codon (Godiker et al., 2018).

*MT-ATP6*, encoding the ATP synthetase (complex V) subunit, and *POLG*, encoding the catalytic subunit of polymerase gamma, are essential for mitochondrial biogenesis through OXPHOS and DNA replication mechanisms, respectively. In the complex V subunit encoded by *MT-ATP6*, the pathogenic variants at m.8993 are involved in nearly 50% of the reported *MT-ATP6* disease cases. The m.8993T>C variant causes a milder disease phenotype than m.8993T>G (Ganetzky et al., 2019). The *MT-ATP6* gene variant (m.8993T>C) was identified in P13, 11 years old, and P14, 13 years old. Both were found to be 99% homoplasmic in their blood cells and shared mild phenotypes; also, the onset of disease and laboratory and imaging findings were similar. Both cases are Leigh-like phenotype with post-infectious acute onset ataxia at 6–7 years, loss of muscle strength, and basal ganglia involvement in cranial MRI. Their symptoms were alleviated with mitochondrial support treatments, and they were followed up in the metabolism outpatient clinic.

The *POLG* mutations, which are clinically among the mitochondrial depletion syndromes, are characterized by quantitative abnormalities of the mitochondrial DNA, causing the impairment of energy production in single or multiple organ systems (Rahman and Poulton, 2009). Myopathy may cause symptoms from the infantile period to early childhood, and children are primarily brought with complaints of skeletal muscle weakness. The most common symptoms of mitochondrial muscle disease are lethargy, fatigue, exercise intolerance, and pain. Myopathy is generally a part of a complex multisystem disorder in childhood; isolated muscle involvement is a more common characteristic of adult-onset disease. Outer eye muscles are frequently involved in the pediatric group when myopathy is the only finding; myopathy is proximal rather than distal. Many patients with chronic progressive external ophthalmoplegia (CPEO) develop myopathy. P3 and 4, the two affected siblings carrying the *POLG* variant (c.911T>G), initially presented with progressive myopathy that started with the involvement of ophthalmic muscles at 6 years of age in both average growth and neuromotor development. Unlike in the literature data, symptoms were of the early-onset and progressive type. It has been reported that environmental factors such as immunity dysfunction, viral infection, mitochondrial toxins, and genetic modifiers have consequences on the severity of the disease and complicate foresight for patients (Rahman and Copeland, 2019). Both patients 3 and 4 passed away at the age of 17 due to respiratory failure.

### Metabolism/nucleotide metabolism (*TK2*)

Another mitochondrial DNA (mtDNA) depletion syndrome is associated with the *TK2* gene. Thymidine kinase-2 is the first enzyme in the deoxypyrimidine salvage pathway within mitochondria that phosphorylates thymidine and deoxycytidine. These are subsequently converted to deoxynucleoside triphosphates required for mtDNA replication and maintenance. Most patients with early onset before 2 years of age show severe and rapid progression. The less severe forms begin in childhood through adulthood and exhibit slower progression; bulbar, proximal limb, and respiratory weakness can be severe, causing the inability to walk or breathe independently. Early and accurate diagnosis is critical since a specific treatment is under development (Dominguez-Gonzalez et al., 2019; Dominguez-Gonzalez et al., 2022). Two affected siblings from the late-onset group, patient 1, young adult, and patient 2, adolescent, with severe neuromuscular weakness, including diaphragmatic weakness, requiring respiratory support, have, with previously reported homozygous c.323C>T/p.(T108M), a variant in the *TK2* gene. They were able to reach early initiation of medication. The beneficial effects of the therapy were verified by functional tests, including the 6-min walk test, forced vital capacity, and maximal inspiratory pressure, with mean changes that appear to be clinically significant.

### Metabolism/carbohydrate metabolism (*OXCT1*)

The *OXCT1* gene encodes a critical mitochondrial enzyme essential for ketone body metabolism in all extrahepatic tissues (Grunert et al., 2021). P10 with the compound heterozygous variant in the *OXCT1* gene (c.[1173-139G>T];[1370C>T]/p.([?];[T457I]) was a 1.5-year-old baby boy, had normal development until the 11th month, except for lack of postnatal crying and 8 days of admittance to the neonatal intensive care unit. He had his first metabolic attack during an upper respiratory tract infection in the 11th month and two more episodes after gastroenteritis. He had metabolic acidosis; organic acidemia and keratolysis were considered, but a definitive diagnosis could not be achieved. He had arrest during the second attack and responded to resuscitation. He was discharged with a tracheostomy. Metabolic attacks following infection, ketone positivity in blood and urine, and asymptomatic state between attacks all supported succinyl-CoA 3-ketoacid-CoA transferase (SCOT) deficiency, and the compound heterozygosity for the *OXCT1* gene complied with the autosomal recessive inheritance (Sasai et al., 2017). The *OXCT1* gene is associated with SCOT deficiency and causes mitochondrial dysfunction secondary to ketone body catabolism disorder. Manifestation is characterized by clinical findings that depend on mutation intensity and residual enzyme activity, episodic or permanent ketosis.

### Metabolism/fatty acid elongation *(ECHS1)*

The *ECHS1* gene encodes an enzyme to hydrate medium- and short-chained fatty enoyl-CoA thioesters from four carbons long to up to C16, resulting in fatty acid oxidation disorder when defective (Catania et al., 2018). P10 and P11, 12 years and ten years old brothers were offsprings from a nonconsanguineous marriage; both were followed up for ten years without a diagnosis. Their symptoms were a progressive neuromotor developmental delay, spasticity, truncal hypotonia and basal ganglion involvement in cranial MRI. Metabolic and molecular tests were not informative. Two different nonsynonymous variants (c.202G>A/p.(E68K) and c.161G>A/p.(R54H) in the *ECHS1* gene have been identified in these siblings in compound heterozygous form. The c.161G>A alteration was reported in a patient with mitochondrial short-chain enoyl-CoA hydratase-1 deficiency (Fitzsimons et al., 2018). However, the c.202G>A (rs1276839756) variant was not associated before and was reported as very rare in populations (0.0008%) with a “pathogenic” classification. The association of the very first ECHS1 deficiency in MD was presented in a study of 435 individuals with impaired mitochondrial energy metabolism, revealing ten patients with 13 *ECHS1* gene variants, 12 being missense type, suggesting bi-allelic truncating variants may embryonically be lethal. Furthermore, the study suggested that patients may benefit from dietary therapy (Haack et al., 2015).

Limb-girdle muscular dystrophy (LGMD) is a disorder that must be highly considered in the differential diagnosis of MDs due to clinical overlaps. Since not only disrupted mechano-signaling components but also functional insufficiencies of mitochondria play a role, all are rare forms with different inheritance patterns and are genetically quite heterogeneous. The fine detail is the presence of myopathy in the histopathological analysis of the muscle biopsy material and the overlapping in neuromuscular findings in clinical evaluation (Wang et al., 2018). Similar to those observed in mitochondrial depletion, in LGMD, the age of onset varies from childhood to adolescence, and the clinical course is generally progressive (Nigro and Savarese, 2014). An example of its similarities with mitochondrial pathology was revealed in a cohort study composed of dysferlinopathy patients who reported a decrease in the mtDNA copy number, cytochrome c oxidase (COX) deficiency, and a decrease in complex I and IV activities (Liu et al., 2016). Out of the 12 families, 4 (25%) were diagnosed with LGMD.

Accurate diagnosis in clinical science is the fundamental component of timely and effective care and is the basis for improving the patient’s health and providing proper counseling to the families. The discovery of new genes that play a role in mitochondrial pathogenesis increases over time and improves knowledge of cellular homeostasis and metabolism pathways. Non-invasive, bi-genomic sequencing in a single test method has changed the algorithm investigating mitochondrial diseases as the step before an invasive procedure. Today, biochemical and pathologic studies requiring muscle biopsy are carried out when the diagnosis is indistinct, following high-throughput analysis of the nuclear and mitochondrial genome analysis. In the last decade, the concept of MDs has been re-visited, and a new classification is recommended that focuses on genetic origin rather than biochemical deficiency (Ferreira et al., 2021). The mitochondrial and exome sequencing of the patients in our study achieved 67% (16/24) diagnostic success and eight of our families remained undiagnosed. Recent reports with similar approaches have reported different rates of diagnoses, revealing it to be between 39% and 77% (Taylor et al., 2014; Wortmann et al., 2015; Pronicka et al., 2016; Puusepp et al., 2018; Theunissen et al., 2018; Kerr et al., 2020; Kose et al., 2021) (Table 3). In these studies, we observe that the diagnosis rate increases when the MD criterion scale is used. Nevertheless, unknown functional effects of genes or new variants may result in unexplored patients remaining undiagnosed.

**TABLE 3:** Overview of the diagnostic rates of mitochondrial disease by exome sequencing in our study and in other published data.

Criteria	Probands (n)	MD-related genes in gDNA/mtDNA	Nuclear genes unrelated to MDs	ND	Diagnostic yield	Ref. no.
Bi-genomic DNA sequencing	24	9/3	4	8	16/24 (67%)	This study
Two-step bi-genomic DNA sequencing	117	38/23	19	37	80/117 (68%)	39
Traditional diagnosis/genetic, out of 360 patients	116	26/1	26	63	53/116 (45.70%)	40
	—					
	115					
Genetic first	28	1/4	11	12	16/28 (57%)	41
Genetic testing of traditionally diagnosed patients	53	32/-	-	21	32/53 (40%)	42
Genetic first	113	42/6	19	46	67/113 (59.3%)	43
MD criterion scale	59	29/-	17	13	46/59 (77%)	44
MD criterion scale	109	20/1	21	67	42/109 (39%)	45
	42^a^	15/1^a^	8^a^			
	44^b^	4^b^	6^b^			
	23^c^	1^c^	7^c^			

MDs: mitochondrial diseases; ND: not diagnosed.

^a^ High suspicion.

^b^ Intermediate suspicion.

^c^ Low suspicion.

The most efficient approach for diagnosing MDs today is sequencing the nuclear genome and mtDNA if harbored in a single test procedure; however, as evidenced by our study, nuclear gene analysis must be prioritized for diagnostic utility. Investigation of the non-coding regions of the genome by whole-genome sequencing, pathogenic copy number variation in the nuclear genome, and gross deletions or duplications in the mtDNA are the investigations that could not be performed in our study. Furthermore, since mtDNA investigation was conducted on isolated peripheral blood sample cells (P15 with the *MT-TL1* mutation was investigated in both muscle and blood samples), mtDNA variants of low heteroplasmy was not diagnosed. The discipline of mitochondrial medicine has significantly progressed by determining more than 400 genes in the nuclear and mtDNA genomes related to MDs; however, reasons for clinical variations in individuals with the same genetic deficits and tissue specificity remain unanswered.

## Data Availability

The datasets presented in this study can be found in online repositories. The names of the repository/repositories and accession number(s) can be found at: https://www.ncbi.nlm.nih.gov/snp/, SCV001423134.1 https://www.ncbi.nlm.nih.gov/snp/, SCV001423135.1 https://www.ncbi.nlm.nih.gov/snp/, SCV001571651.1 https://www.ncbi.nlm.nih.gov/snp/, SCV001571652.1 https://www.ncbi.nlm.nih.gov/snp/, SCV001571649.1 https://www.ncbi.nlm.nih.gov/snp/, SCV003804637 https://www.ncbi.nlm.nih.gov/snp/, SCV003804638 https://www.ncbi.nlm.nih.gov/snp/, SCV001571647.1 https://www.ncbi.nlm.nih.gov/snp/, and SCV001571646.1.
